# In vitro ablation rates of Ho:YAG, p-Tm:YAG and TFL lasers

**DOI:** 10.1007/s00345-025-06001-9

**Published:** 2025-11-10

**Authors:** A. Quarà, A. Bravo-Balado, S. Moretto, A. Madden, F. Zorzi, L. M. I. Jannello, S. Kutchukian, J. Cabrera, M. Corrales, M. Chicaud, U. Gradilone, L. Candela, C. Gorny, F. Coste, L. Berthe, S. Doizi, F. Panthier, C. Fiori, O. Traxer

**Affiliations:** 1 GRC n°20, Groupe de Recherche Clinique sur la Lithiase Urinaire, Hôpital Tenon, Sorbonne Université, Paris, 75020 France; 2 Service d’Urologie, Assistance-Publique Hôpitaux de Paris, Hôpital Tenon, Sorbonne Université, 4 rue de la Chine, Paris, 75020 France; 3 Division of Urology, Department of Oncology, University of Turin, San Luigi Gonzaga Hospital, Orbassano, Italy; 4 PIMM, UMR 8006 CNRS-Arts et Métiers ParisTech, 151 bd de l’Hôpital, Paris, 75013 France; 5 Department of Urology, Humanitas Clinical and Research Institute IRCCS, Milan, Italy; 6 Urology Clinic, Department of Medical, Surgical and Health Sciences, University of Trieste, Trieste, 34127 Italy; 7 Università degli Studi di Milano, Milan, Italy; 8 Department of Urology, Foundation IRCCS Ca’ Granda, Ospedale Maggiore Policlinico, Milan, 20122 Italy; 9 Poitiers University hospital, Poitiers, France; 10 Service d’urologie, CHU de Limoges, 2 avenue Martin Luther King, Limoges, 87000 France

**Keywords:** Laser lithotripsy, Ho:YAG, Pulsed Thulium:YAG laser, Thulium fiber laser, Ablation, Kidney stones

## Abstract

**Objective:**

This study compares the ablation rates of three laser systems—Holmium:YAG (Ho:YAG), Thulium Fiber Laser (TFL), and Pulsed Thulium:YAG (p-Tm:YAG)—for renal stone lithotripsy using a standardized robotic setup.

**Materials and methods:**

A robotic arm enabled consistent laser application on stone phantoms simulating calcium oxalate monohydrate (hard) and uric acid (soft) stones. Ablation efficiency (mm³/J) was assessed across different laser settings (0.2 J–50 Hz, 0.5 J–20 Hz, and 1.0 J–10 Hz) and fiber diameters (200 and 272 μm). Ablated volumes were quantified via micro-CT and 3D segmentation using 3DSlicer. Statistical analysis evaluated differences in performance.

**Results:**

TFL demonstrated the highest ablation rates for both hard and soft stones, significantly outperforming Ho:YAG in multiple settings. For hard stones, TFL exhibited greater ablation efficiency than Ho:YAG, particularly at 0.5 J–20 Hz and 1.0 J–10 Hz. The p-Tm:YAG laser also outperformed Ho:YAG at 0.5 J–20 Hz. For soft stones, the difference between TFL and Tm:YAG was statistically significant at lower energy settings (0.20 J–50 Hz and 0.5 J–20 Hz). Compared to Ho:YAG, TFL showed significantly higher ablation rates across all tested settings (*p* < 0.05). The p-Tm:YAG laser showed intermediate performance, with higher efficiency than Ho:YAG but slightly lower than TFL. Fiber diameter influenced ablation, with 272 μm fibers yielding greater efficiency at lower energy settings (*p* < 0.05 at 0.20 J − 50 Hz and 0.5 J − 20 Hz for both stone types); this comparison was limited to p-Tm:YAG, as data for the other lasers are already available in the literature.

**Conclusion:**

TFL achieved the highest in vitro ablation efficiency. However, p-Tm:YAG represents a promising compromise, offering improved performance over Ho:YAG and a balanced profile between fragmentation and dusting capabilities.

## Introduction

Laser lithotripsy has revolutionized the management of urinary stones, offering minimally invasive solutions with high efficacy and safety. Over the years, Holmium: Yttrium-Aluminum-Garnet (Ho:YAG) has been the gold standard for laser lithotripsy, demonstrating reliability in stone fragmentation and dusting [1–3].

However, recent advancements in laser technology have introduced alternative systems, such as the Thulium Fiber Laser (TFL) and the Pulsed Thulium: Yttrium-Aluminum-Garnet (p-Tm:YAG) laser, which offer distinct physical properties that may enhance stone ablation efficiency and procedural outcomes [4].

TFL has gained significant attention due to its high repetition rates, lower pulse energy and improved fragmentation efficiency, which allow for more effective pulverization strategies and reduced retropulsion, without the need to increase the frequency for faster ablation [5–7]. In contrast, the newly developed p-Tm:YAG laser provides an intermediate profile between Ho:YAG and TFL, with a balanced combination of peak power (PP), pulse duration, and ablation performance [8]. Although these technologies have been individually studied, direct comparisons under standardized conditions remain limited [9].

A direct comparison between TFL and Ho:YAG has been recently conducted using a standardized robotic arm to ensure highly reproducible and controlled experimental conditions. Building on this methodology, the present study expands the comparison by incorporating pulsed Tm:YAG. Notably, two p-Tm:YAG systems are currently available on the market: the Dornier Thulium, for which several in vitro and in vivo studies exist, and the Omniguide Revolt, for which data remain scarce [10].

This study aims to provide a comprehensive evaluation of the ablation rates of Ho:YAG, TFL, and p-Tm:YAG lasers for lithotripsy using a controlled and reproducible experimental setup. By analysing their performance across different stone compositions and laser settings, we seek to clarify the advantages and limitations of each laser technology and contribute to the optimisation of laser lithotripsy techniques to improve clinical outcomes.

## Materials and methods

### Ho:YAG, TFL, and p-Tm:YAG generators and fibers

In this study, we did compared the performance of three different laser sources for medical applications. The first device tested was a 50 W TFL (IPG Photonics^®^, Russia) with a wavelength of 1940 nm, used a 272 μm fibers [11]. The second system was a diode-pumped solid-state (DPSS) p-Tm:YAG RevoLix HTL prototype (OmniGuide, USA) laser with a wavelength of 2013 nm, tested with both 200 μm and 272 μm fibers. Finally, we evaluated the performance of a 30 W Ho:YAG laser (MH1 Rocamed^®^, Monaco) with a wavelength of 2120 nm, also coupled with 272 μm fibers.

### Stone phantoms

BegoStones measuring 1 cm³ were fabricated following previously described methods [12]. To replicate calcium oxalate monohydrate (Hard) and uric acid (Soft) stones, we used a “powder-to-water” ratio of 15:3 for Hard stones and 15:5 for Soft stones. After shaping, the samples underwent a 48-hour drying period at 30 °C to reduce heterogeneity.

### Experimental setup

Stone phantoms were fully submerged in saline solution at room temperature and securely fixed within a bench model. To evaluate the performance of the TFL, p-Tm:YAG, and Ho:YAG lasers, we used 272 μm fibers. Additionally, the ablation efficiency of the p-Tm:YAG laser was also assessed using a 200 μm fiber. The laser fiber tip was positioned perpendicularly and kept in direct contact with the stone surface. A custom-designed fiber support was developed to ensure complete stability during laser emission. To minimize the “burnback effect”, we adopted a “one locus-one pulse” approach [13]. A six-axis robotic arm (KR6R900, Kuka International ©, Germany) was employed to execute a precisely programmed spiral trajectory.

Laser emission and robotic arm movement were synchronized and controlled via computational command. The laser fiber followed an Archimedean Type-4 spiral trajectory for 20 s, with a 4 mm radius and a constant 1.2 mm spacing between spiral turns. Regarding laser settings, three different configurations were tested for all laser types: 0.2 J–50 Hz, 0.5 J–20 Hz, and 1 J–10 Hz. While TFL was operated in short pulse mode, the Ho:YAG were set to long pulse mode and the p-Tm:YAG laser was set to 50%.

**Fig. 1:**
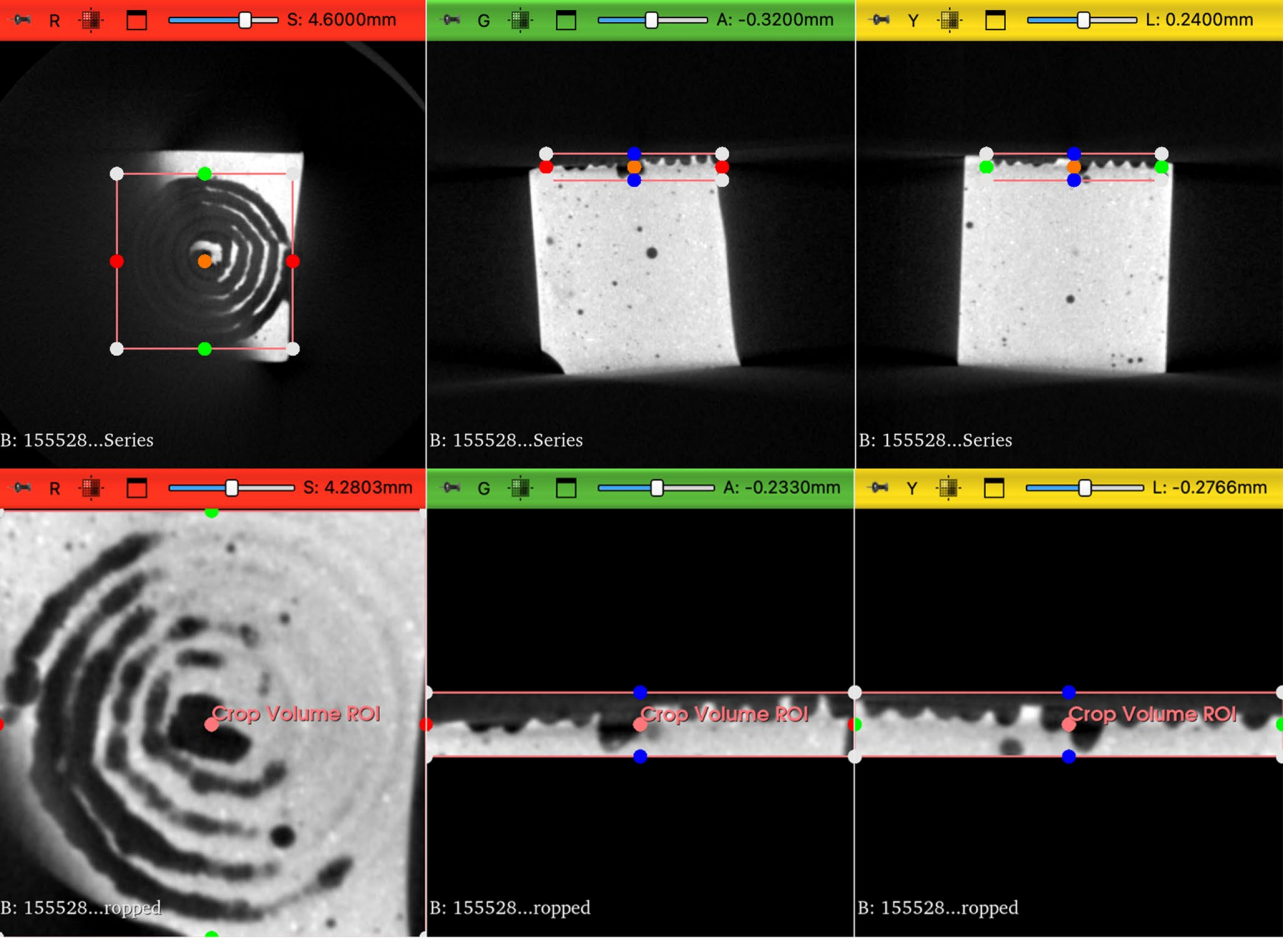
3DSlicer segmentation method to evaluate ablation volumes: stone segmentation from CT by selecting the region of interest (ROI)

**Fig. 2:**
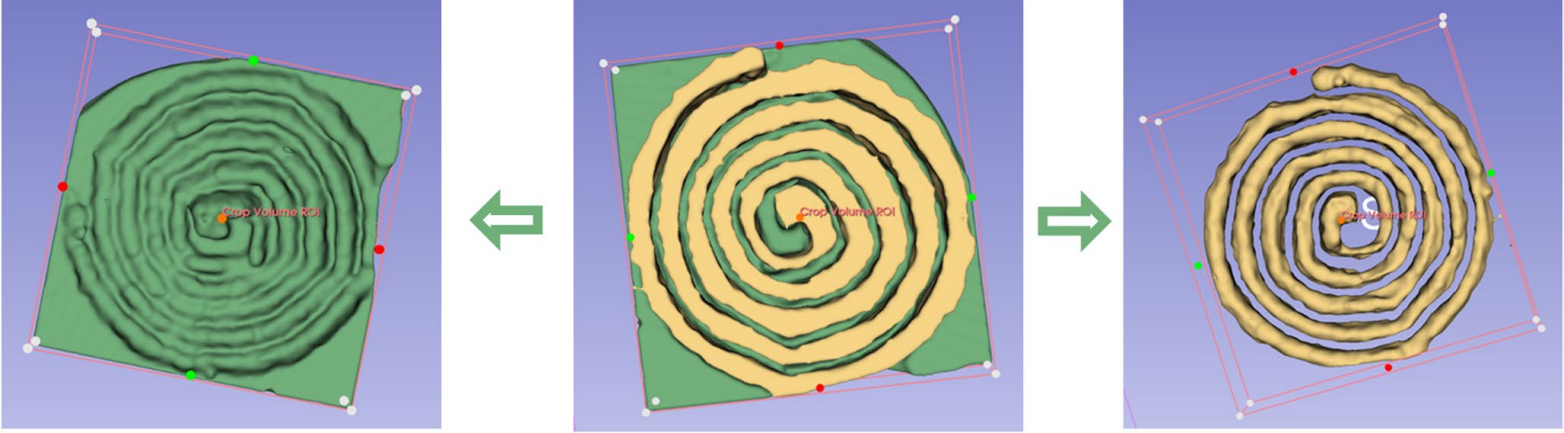
3DSlicer segmentation method for evaluating ablation volumes: segmentation of the spiral crater area and compilation of both to obtain the ablation volume

Each test was performed in three times to ensure reproducibility. Following laser emission, the stones were dried according to previously described protocols. Three-dimensional imaging of the artificial stones was conducted using a micro-CT scanner (Quantum FX, Perkin Elmer©), and volumetric analysis was carried out through 3D segmentation using 3DSlicer software (NIH©) [11] (Figs. 1 and 2). All volumetric analyses were reviewed by a single operator (A.Q.) to eliminate inter-individual variability.

### Statistical analysis

For the analysis of ablation rates, a two-tailed Student’s t-test was performed using IBM SPSS Statistics for Macintosh, Version 29.0.2.0 (IBM Corp., Armonk, NY, 2023). A p-value of less than 0.05 was considered statistically significant.

## Results

The comparison of ablation rates among the Ho:YAG, TFL, and p-Tm:YAG lasers revealed significant differences in performance depending on the laser type, energy settings, and stone composition.

For hard stones, the TFL laser consistently demonstrated absolute higher ablation rates than both the p-Tm:YAG and Ho:YAG lasers at all energy settings. Although the difference between TFL and p-Tm:YAG was not statistically significant (*p* > 0.05), the TFL exhibited significantly greater ablation efficiency than the Ho:YAG laser, particularly at 0.5 J–20 Hz (27.90 mm^3^/min ± 8.83 vs. 16.84 mm^3^/min ± 1.17 - *p* = 0.04) and 1.0 J–10 Hz (29.53 mm^3^/min ± 3.66 vs. 20.86 mm^3^/min ± 1.76 - *p* = 0.01). The p-Tm:YAG laser also outperformed the Ho:YAG laser at 0.5 J–20 Hz (22.96 mm^3^/min ± 3.03 vs. 16.84 ± 1.17 - *p* = 0.01), but no significant difference was observed at 1.0 J–10 Hz (25.02 mm^3^/min ± 6.42 vs. 20.86 mm^3^/min ± 1.76 - *p* = 0.17) (Tables 1, 2 and 3).

For soft stones, the TFL laser demonstrated superior ablation rates compared to both the p-Tm:YAG and Ho:YAG lasers. The difference between TFL and Tm:YAG was statistically significant at lower energy settings, with p-values of 0.03 (30.83 mm^3^/min ± 3.55 vs. 25.76 mm^3^/min ± 0.17) and 0.00 (32.65 mm^3^/min ± 1.76 vs. 24.95 mm^3^/min ± 0.90) for 0.20 J–50 Hz and 0.5 J–20 Hz, respectively. In the comparison between TFL and Ho:YAG, TFL showed significantly higher ablation rates at all tested energy settings (*p* < 0.05). Additionally, p-Tm:YAG outperformed Ho:YAG for soft stones at 0.5 J–20 Hz and 1.0 J–10 Hz, though the differences were not statistically significant (*p* = 0.08 for both) (Tables 1, 2 and 3).

Finally, the comparison of ablation efficiency between 200 μm and 272 μm fibers using the Tm:YAG laser showed that the 272 μm fiber resulted in significantly higher ablation rates for both hard and soft stones at lower energy settings. Statistically significant differences were observed at 0.20 J–50 Hz and 0.5 J–20 Hz for both stone types (Table 4).

## Discussion

This study provides a comprehensive comparison of TFL, p-Tm:YAG, and Ho:YAG lasers for kidney stone ablation using a standardized robotic arm. Notably, this is the first comparative study to incorporate a fully automated mechanical robotic arm, ensuring precise and reproducible conditions for laser evaluation.

Our results demonstrate that p-Tm:YAG achieves excellent ablation efficiency across the two stone-phantom compositions designed to mimic calcium oxalate monohydrate (COM) and uric-acid (UA) stones. These findings are in line with earlier work showing that p-Tm:YAG effectively ablates both COM and UA stones without statistically significant differences, confirming its robust performance irrespective of stone composition or laser settings [14].

Importantly, we acknowledge that the definition and fabrication of our phantoms are grounded in their acoustical—rather than optical—characteristics, following the approach described by Esch et al., which matches the elastic and fracture properties that dominate fragmentation dynamics [12].

Early clinical experiences have demonstrated the effectiveness and safety of Dornier p-Tm:YAG in flexible ureteroscopic lithotripsy, yielding comparable stone-free rates to TFL (75% vs. 77%, *p* = 0.8), albeit with a slightly lower dusting efficiency [15, 16].

Recent systematic reviews confirm that p-Tm:YAG is a viable alternative to Ho:YAG and TFL, offering a balance between these technologies for endoscopic lithotripsy applications. Additionally, purely p-Tm:YAG lasers have shown promising safety and efficacy in RIRS and PCNL, with their higher PP compared to TFL enabling efficient stone disintegration and effective pulverization [17, 18].

Our findings indicate that for hard stones, TFL consistently demonstrated higher ablation rates than both Tm:YAG and Ho:YAG, regardless of energy settings. The difference between TFL and Tm:YAG was not statistically significant, however, TFL significantly outperformed Ho:YAG at 0.5 J–20 Hz and 1.0 J–10 Hz.

While both p-Tm:YAG and TFL outperformed Ho:YAG, a key distinction emerged in their dusting efficiency. A recent study shows that p-Tm:YAG produced lower zero fragmentation rates (ZFR) than TFL (39% vs. 64%, *p* = 0.008), suggesting a relatively low ability to generate particulate matter [16]. This implies that TFL remains superior for dusting strategies, whereas p-Tm:YAG maintains a balanced profile between fragmentation and dusting [16]. Notably, Tm:YAG also exhibited higher efficiency than Ho:YAG at 0.5 J–20 Hz (*p* = 0.01). These results suggest that while TFL is the most effective option for hard stone ablation, Tm:YAG still provides significant advantages over Ho:YAG, particularly at certain energy settings.

For soft stones, TFL demonstrated superior ablation rates compared to both Tm:YAG and Ho:YAG, with statistically significant differences at lower energy settings. Additionally, the comparison between TFL and Ho:YAG confirmed TFL’s superior performance, as it achieved significantly higher ablation rates across all tested energy settings (*p* < 0.05). Although Tm:YAG outperformed Ho:YAG at 0.5 J–20 Hz and 1.0 J–10 Hz, these differences were not statistically significant (*p* = 0.08 for both).

These findings align with recent literature. A recent in vitro study by Sierra del Rio et al., reported that both p-Tm:YAG and TFL exhibited superior performance in laser lithotripsy compared to Ho:YAG, demonstrating higher efficiency and ablation speed. Moreover, thermal damage was not linked to a specific laser type but was instead associated with increasing power settings [8]. Additionally, a recent study by Petzold et al. showed that longer pulse durations significantly enhance Tm:YAG’s ablation rates compared to Ho:YAG, further supporting its efficacy [19].

From a technical perspective, the p-Tm:YAG laser represents an intermediate option between Ho:YAG and TFL, balancing key properties such as PP, a uniform pulse profile, and reduced risk of fiber fracture in vitro. The lower PP and more uniform pulse profile of both TFL and p-Tm:YAG play a major role in reducing retropulsion, allowing for prolonged contact between the fiber tip and the stone. This extended interaction enhances fragmentation efficiency, positioning both p-Tm:YAG and TFL as superior to Ho:YAG in terms of ablation performance [20–23].

It is important to underline that our experiments were performed exclusively with the Revolix pulsed Tm:YAG system. Another pulsed Tm:YAG laser, the Thulio^®^, is also available on the market and is increasingly used in many centers. These two devices present notable differences, therefore, our results should not be generalized to all pulsed Tm:YAG systems, and future studies directly comparing RevoLix HTL^®^ and Thulio^®^ would be of great interest to further clarify the similarities and differences between these technologies [24].

Regarding the comparison between fiber diameters, this analysis was performed only with the p-Tm:YAG laser. We chose to explore this aspect because comparative data on fiber size for Ho:YAG and TFL are already well-documented in the literature. Nevertheless, we agree that the main focus of the present study is the comparison among the three laser technologies, and therefore we now present the fiber-size results as a complementary discussion point rather than as part of the primary results. This adjustment clarifies that the fiber analysis is exploratory in nature and secondary to the core comparison of laser systems.

Finally, our study demonstrated that using a 272 μm fiber with the Tm:YAG laser resulted in significantly higher ablation rates than the 200 μm fiber for both hard and soft stones at lower energy settings, with statistically significant differences observed at 0.20 J–50 Hz and 0.5 J–20 Hz. These findings are consistent with those reported by Panthier et al. [10] for TFL and Ho:YAG, reinforcing the importance of fiber diameter in optimizing laser lithotripsy efficiency [10]. In clinical practice, however, smaller fibers are commonly used with TFL, typically 150 μm, differing from the larger diameters often employed with other lasers.

Despite its novelty, the current study has several limitations. BegoStones phantoms are widely used in vitro due to their consistency and ease of handling, however, they do not fully replicate the optical absorption and thermal properties of human stones. Consequently, laser settings effective on BegoStones phantoms may not yield identical results in clinical practice due to differences in absorption, fragmentation patterns, and thermal interactions. Nonetheless, their use provides a controlled and reproducible model for evaluating laser performance.

It is also important to underline that our model does not allow for a detailed analysis of fragment size or composition, since only fine dust is generated with stone phantoms. As a result, we were unable to investigate the fragmentation pattern or potential for clinically relevant fragments, which is a crucial outcome when treating real human stones. In vivo studies could provide valuable insights into this aspect, as has already been shown for Thulium Fiber Laser [14].

**Table 1:** Comparison between TFL and Tm:YAG lasers (Results shown in bold indicate statistical significance (p < 0.05))

Interface	Laser settings	Ablation rate (mm^3^/min)		p value
		TFL (mean ± SD)	p-Tm:YAG (mean ± SD)	
Hard Stones	0.20 J–50 Hz	25.95 (± 7.35)	19.74 (± 1.16)	0.11
	0.5 J–20 Hz	27.90 (± 8.83)	22.96 (± 3.03)	0.21
	1.0 J–10 Hz	29.53 (± 3.66)	25.02 (± 6.42)	0.18
Soft stones	0.20 J–50 Hz	30.83 (± 3.55)	25.76 (± 0.17)	**0.03**
	0.5 J–20 Hz	32.65 (± 1.76)	24.95 (± 0.90)	**0.00**
	1.0 J–10 Hz	33.64 (± 5.19)	27.16 (± 8.43)	0.16

**Table 2:** Comparison between TFL and Ho:YAG lasers (Results shown in bold indicate statistical significance (p < 0.05))

Interface	Laser settings	Ablation rate (mm^3^/min)		p value
		TFL (mean ± SD)	Ho:YAG (mean ± SD)	
Hard stones	0.20 J–50 Hz	25.95 (± 7.35)	NA	NA
	0.5 J–20 Hz	27.90 (± 8.83)	16.84 (± 1.17)	**0.04**
	1.0 J–10 Hz	29.53 (± 3.66)	20.86 (± 1.76)	**0.01**
Soft stones	0.20 J–50 Hz	30.83 (± 3.55)	NA	NA
	0.5 J–20 Hz	32.65 (± 1.76)	16.57 (± 8.38)	**0.02**
	1.0 J–10 Hz	33.64 (± 5.19)	18.57 (± 3.03)	**0.00**

**Table 3:** Comparison between Ho:YAG and Tm:YAG lasers (Results shown in bold indicate statistical significance (p < 0.05))

Interface	Laser settings	Ablation rate (mm^3^/min)		p value
		p-Tm:YAG (mean ± SD)	Ho:YAG (mean ± SD)	
Hard stones	0.20 J–50 Hz	19.74 (± 1.16)	NA	NA
	0.5 J–20 Hz	22.96 (± 3.03)	16.84 (± 1.17)	**0.01**
	1.0 J–10 Hz	25.02 (± 6.42)	20.86 (± 1.76)	0.17
Soft stones	0.20 J–50 Hz	25.76 (± 0.17)	NA	NA
	0.5 J–20 Hz	24.95 (± 0.90)	16.57 (± 8.38)	0.08
	1.0 J–10 Hz	27.16 (± 8.43)	18.57 (± 3.03)	0.08

**Table 4:** Comparison of 200 μm vs. 272 μm fibers in Tm:YAG laser (Results shown in bold indicate statistical significance (p < 0.05))

Interface	Laser settings	Ablation rate (mm^3^/min)		p value
		200 μm (mean ± SD)	272 μm (mean ± SD)	
Hard stones	0.20 J–50 Hz	15.93 (± 0.83)	19.74 (± 1.16)	**0.00**
	0.5 J–20 Hz	17.09 (± 1.58)	22.96 (± 3.03)	**0.02**
	1.0 J–10 Hz	19.30 (± 0.50)	25.02 (± 6.42)	0.10
Soft stones	0.20 J–50 Hz	20.65 (± 1.92)	25.76 (± 0.17)	**0.01**
	0.5 J–20 Hz	22.39 (± 0.84)	24.95 (± 0.90)	**0.01**
	1.0 J–10 Hz	22.72 (± 3.56)	27.16 (± 8.43)	0.22

Moreover, although this study focuses on ablation rate as a primary endpoint, it should be noted that in clinical practice the ultimate success criteria are stone-free rates (SFR) and the need for retreatment, which depend not only on ablation efficiency but also on fragmentation characteristics, retropulsion, and the ability to clear residual fragments [25].

From a mechanistic standpoint, while it is generally accepted that stone ablation occurs through laser-induced energy absorption by the stone surface, the dominant underlying mechanism, whether photothermal or photomechanical, remains a matter of ongoing investigation. It is currently hypothesized that thermal effects play a primary role, particularly with TFL and p-Tm:YAG lasers due to their lower peak power and longer pulse duration, which promote gradual energy deposition and heat-driven disintegration. Some studies have contributed significantly to this understanding and support the predominance of thermal mechanisms, although the interplay with photomechanical forces cannot be excluded [26].

Finally, it should be acknowledged that only one representative device was tested for each laser category (Ho:YAG, TFL, and p-Tm:YAG). Since multiple commercial systems exist with specific technical characteristics (e.g., peak power, pulse modulation, fiber compatibility, noise emission), our findings cannot be generalized to all devices within the same laser family [27]. Together with the intrinsic limitations of the in vitro design, which does not replicate irrigation dynamics, anatomical variability, or patient-related factors, this further underlines the need for complementary in vivo studies to validate and expand our results [28].

## Conclusion

TFL confirmed to be the most effective laser for in vitro lithotripsy, while p-Tm:YAG provides a promising middle-ground solution. Indeed, p-Tm:YAG represents a viable alternative to Ho:YAG, offering a balance between fragmentation and dusting efficiency.

## Data Availability

No datasets were generated or analysed during the current study.
